# Inflammation Determines the Capacity of Allogenic Endothelial Cells to Regulate Human Treg Expansion

**DOI:** 10.3389/fimmu.2021.666531

**Published:** 2021-07-09

**Authors:** Amy Rachael Cross, Julien Lion, Karine Poussin, Denis Glotz, Nuala Mooney

**Affiliations:** ^1^ Human Immunology, Pathophysiology and Immunotherapy, INSERM U976, Paris, France; ^2^ Université de Paris, INSERM U976, Paris, France; ^3^ Service de Néphrologie et Transplantation, Hôpital Saint Louis, Paris, France

**Keywords:** organ transplantation, endothelial cell, Treg, HLA class II, allogenicity, inflammation

## Abstract

During allotransplantation, the endothelium acts as semi-professional antigen-presenting cells with the ability to activate proliferation and to promote differentiation of CD4^+^-T subsets. These abilities are dependent on the luminal expression of HLA class II antigens by microvascular endothelial cells, which is regulated by inflammatory cytokines. The upregulation of HLA-DR and HLA-DQ during rejection implies significant intragraft inflammation. Furthermore, the microvascular inflammation is an independent determinant for renal allograft failure. In this study, the potential of inflammation to modify endothelial regulation of peripheral CD4^+^ Treg cells was examined. Microvascular endothelial cells were exposed to pro-inflammatory cytokines for varying durations before co-culture with PBMC from non-HLA matched donors. Proliferation and expansion of CD4^+^Treg and soluble factor secretion was determined. Early interactions were detected by phosphorylation of Akt. Video microscopy was used to examine spatial and temporal endothelial-CD4^+^T interactions. Highly inflammatory conditions led to increased endothelial expression of HLA-DR, the adhesion molecule ICAM-1, the costimulatory molecule PD-L1 and *de novo* expression of HLA-DQ. Treg differentiation was impaired by exposure of endothelial cells to a high level of inflammation. Neither IL-6, IL-2 nor TGFβ were implicated in reducing Treg numbers. High PD-L1 expression interfered with early endothelial cell interactions with CD4^+^T lymphocytes and led to modified TCR signaling. Blocking endothelial PD-L1 resulted in a partial restoration of Treg. The allogenic endothelial cell-mediated expansion of Treg depends on a critical threshold of inflammation. Manipulation of the PD-L1/PD-1 pathway or endothelial activation post-transplantation may promote or interfere with this intrinsic mechanism of allospecific Treg expansion.

## Introduction

After solid organ transplantation, endothelial cells become the primary interface between donor tissues and anti-donor immune responses. However, their immunoregulatory potential has been largely neglected. As semi-professional antigen-presenting cells, the endothelium is capable of modulating anti-donor T helper responses under inflammatory conditions (1, 2). Furthermore, the HLA and costimulatory profiles of the endothelium are modified in the inflammatory milieu of allograft rejection, which may lead to changes in lymphocyte regulation.

Expression of HLA class II antigens is strongly increased following transplantation and is associated with rejection of renal, cardiac and liver allografts (3–6). Healthy endothelium constitutively expresses HLA-DR, whilst HLA-DQ is barely detectable (3–7). Post-transplantation, HLA-DQ becomes readily expressed and easily detectable. *In vitro* culture of microvascular endothelial cells leads to loss of HLA class II expression (7, 8). The constitutive expression of HLA-DR in renal microvascular endothelial cells is dependent upon the MHC Class II transactivator (CIITA) through the IFNγ-dependent promoter IV and basal physiological levels of IFNγ (7). Addition of IFNγ *in vitro* rapidly increases endothelial HLA-DR expression, whilst HLA-DQ requires prolonged stimulation. TNFα alone does not alter either HLA-DR or HLA-DQ expression (8), but the combination of IFNγ and TNFα notably enhances HLA-DQ induction (8, 9). These *in vitro* studies imply the necessity for significant inflammation within the allograft in order to promote HLA-DQ expression.

Between 1 to 10% of T lymphocytes are estimated to recognize allogenic HLA-peptide complexes (10, 11). Strong canonical TCR signaling (through Zap70 and Akt) results in migratory arrest, proliferation and differentiation into effector cells (12). TCR signals are enhanced or antagonized by costimulatory factors and coinhibitory factors (such as PD-L1) (13). The strength of TCR signaling can determine whether naïve CD4^+^ T lymphocytes differentiate into effector cells or regulatory cells (12, 14–16). The regulatory T cell differentiation can be consolidated by cytokines (e.g. IL-2 and TGFβ) and certain costimulatory molecules (17).

Human endothelial cells can induce proliferation of alloreactive memory CD4^+^ T lymphocytes through their expression of HLA class II antigens (18–21). Moreover allogenic microvascular endothelial cells selectively expand pro-inflammatory Th1 and Th17 subsets, as well as anti-inflammatory memory Treg (18, 22). Steady-state production of IL-6 by endothelial cells drives Th17 expansion and increased IL-6 secretion further enhances this differentiation (18, 23). The amplification of memory Treg requires direct contact with endothelial cells and endothelial expression of ICAM-1^18^. Endothelial regulation of T cell polarization is a dynamic process and can be modulated by immunosuppressors (24) or by HLA class II donor-specific antibodies (23, 25). Of note, donor-specific antibodies against HLA-DR and HLA-DQ cooperate to amplify IL-6 secretion and to impair memory Treg numbers (23, 25).

Transcriptional profiling of biopsies revealed increases in both T effector and Treg transcripts during rejection (26). Intragraft infiltration of Th17 is associated with worse allograft survival (27, 28), whilst intragraft Treg associates with tolerance and improved graft survival (27, 29). In murine models, the adoptive transfer of Treg prolongs survival of dermal (30, 31) and cardiac allografts (32). Given the association between Treg and allograft survival, endothelial regulation of Treg alloresponses may play an important role in preventing graft damage.

Rejection is characterized by manifestations of vascular inflammation, such as peritubular capillaritis, glomerulitis, interstitial inflammation, layering of the basement membrane and the formation of endothelial lesions (26, 33, 34). Microvascular inflammation has been repeatedly associated with risk of graft failure (35–37). Moreover, analysis of RNA transcripts from patient biopsies during rejection reveals the selective activation of the endothelium (26, 38). Considering the vascular inflammation and endothelial activation observed during rejection, this study examined the immunoregulatory ability of human endothelial cells exposed to distinct levels of inflammation. The inflammatory conditions required to induce HLA-DQ expression by endothelial cells were identified and the capacity for expansion of functional Treg under such conditions was evaluated.

## Materials and Methods

### Cell Lines and Culture Reagents

Human renal glomerular endothelial cells (HRGEC) were purchased from Innoprot (Spain) and cultured in Endothelial Cell Medium (ScienCell). The microvascular endothelial cell line, HMEC-1, was cultured in supplemented MCDB-131 medium as described (18, 23, 24). Cells were detached by 0.05% trypsin (Life Technologies); Versene (Life Technologies) was used when VE cadherin expression was assessed. Two models of inflammation were studied; endothelial cells either activated with 20ng/ml interferon γ (IFNγ; Eurobio) for three days to produce ‘activated’ endothelial cells (aEC) or activated with 20ng/ml IFNγ for 7 days in addition to 10ng/ml tumor necrosis factor α (TNFα; Peprotech) for days 3-7 to produce ‘highly activated’ endothelial cells (haEC). Expression of HLA-DQ by primary renal endothelial cells (HRGEC) was induced after addition of 40ng/ml IFNγ for 7 days in addition to 20ng/ml TNFα for the final three days of activation as indicated. Use of IFNγ or a combination of IFNγ and TNFα to induce HLA II expression on endothelial cells has been reported^8^.

Blood was obtained from healthy donors in compliance with the institutional regulations of the Etablissement Français du Sang (Paris, France). Peripheral blood mononuclear cells (PBMC) were isolated by Ficoll density gradient separation (Eurobio) and cultured in supplemented RPMI-1640 medium (Life technologies) (23). CD4^+^ T lymphocytes were enriched from whole PBMC by negative selection isolation kits (MACS Miltenyi Biotech).

### Allogenic Endothelial Cell and Non-Matched PBMC Cultures

Endothelial cells (aEC or haEC) were irradiated prior to adding non-HLA-matched PBMC (1:1) and were co-cultured for 7 days before analysis of CD4^+^ T lymphocytes as described (18, 23, 24). The medium was not changed during the co-culture. Soluble factors in the supernatants of co-cultures were collected after 3 days. To assess the role of soluble factors in Treg expansion, aEC or haEC were seeded in 12-well plates and separated from PBMC by a porous cell culture insert (0.4µm pore size; Becton Dickinson). Carboxyfluorescein succinimidyl ester (CFSE; Biolegend) labelling determined the proliferation of memory T cells and Treg. CD4^+^ Treg subsets were identified by flow cytometry: memory Treg (CD4^+^CD45RA^−^FoxP3^high^) and naive Treg (CD4^+^CD45RA^+^FoxP3^+^).

### Blocking PD-L1 and CD54 by Monoclonal Antibodies

Endothelial cells were incubated with 10µg/ml of blocking antibodies (anti-human PD-L1 (29E.2A3; Biolegend), anti-human CD54 (BBlG-l1 (IIC8); R&D Systems) or appropriate isotype controls (mouse IgG2b (MPC-11; Biolegend) or mouse IgG1 (11711; R&D Systems)) at 4°C for 30 minutes, then at 37°C for a further 30 minutes before washing, irradiating and culture with PBMC. Where indicated anti-PD-L1 antibody was left throughout the co-culture.

### Treg Suppression Assays

After an initial culture of endothelial cells and PBMC, memory Treg (CD4^+^CD45RA^−^CD127^-^CD25^high^) and naïve Treg (CD4^+^CD45RA^+^CD127^-^CD25^+^) were sorted (BD FACS Aria II System). CD4^+^ T cells were isolated from autologous PBMC and stained with 5µM CFSE (Biolegend) as per manufacturer’s instructions. Sorted Treg subsets, autologous CFSE-CD4^+^ T cells and Dynabeads^®^ Human T-Activator CD3/CD28 Dynabeads (Life technologies) were cultured for 3 days before cell proliferation was evaluated by flow cytometry.

### Antibodies and Flow Cytometry

Flow cytometry was carried out on a FACS Canto II (BD Biosciences). Endothelial cells were phenotyped with the following antibodies: HLA-DR APC (L243), HLA-DQ PE (HLA-DQ1), HLA-ABC APC/Cy7 (W6/32), VE Cadherin PerCP/Cy5.5 (BV9), PD-L1 PE/Cy7 (29E.2A3), CD59 PE [P282 (H19)], CD55 FITC (JS11) and CD46 PE/Cy7 (TRA-2-10; Biolegend) and ICAM-1 FITC (84H10; Beckman Coulter). Lymphocyte polarization was analyzed using: CD4 PE (RPA-T4; BD Pharmingen), IFN-γ FITC (B27; BD Biosciences), CD3 PerCP (SK7; Becton Dickinson); CD4 PB (RPA-T4), CD8 PB (RPA-T8), CD45RA PE/Cy7 (H100), CD25 PE (M-A251), CD127 PerCP/Cy5.5 (A019D5), CD185 APC/Cy7 (J252D4; Biolegend) and IL-17 efluor660 (eBioscience). FoxP3 was detected using the anti-Human Foxp3 Staining Set (236A/E7; eBioscience). Intracellular staining of HLA-DR and HLA-DQ was carried out on fixed and permeabilised cells (2% PFA (Sigma) and 0.5% saponin (Sigma)). Intracellular staining of phosphorylated Akt was carried out in fixed and permeabilised cells (1.5% PFA followed by pure methanol (Millipore) at -20°C) using the following antibodies pAkt (Ser473) APC (SDRNR) and anti-mouse IgG Alexa Fluor^®^ 647 (Poly4053; Biolegend).

### Cytokine Quantification by Enzyme-Linked Immunosorbent Assay

Enzyme-Linked ImmunoSorbent Assays were performed according to the manufacturer’s protocol for human IL-6 (Biolegend), IL-2 (Biolegend) and TGFβ1 (R&D Systems). Duplicate supernatants were acidified and neutralized to activate latent TGFβ1.

### Time-Lapse Microscopy and Image Analysis

Endothelial cells were seeded at 60,000 cells per quadrant in Hi-Q^4^ culture dishes (Nikon Ibidi). 150,000 CFSE-labelled CD4^+^ T cells were added and allowed to sediment for 30 minutes before image-capture began. Time-lapse microscopy was carried out at a rate of 1 image per minute over 75 minutes at x20 magnification using a Nikon Biostation IM-Q at 37°C and 5% CO_2_.

CellProfiler (39), an image analysis software, was used to identify and track cells. A first pipeline was constructed to manually define endothelial cell occupied space and produce a series of masks based on every 10^th^ phase contrast image. A second pipeline identified T cells, measured cell characteristics and related the position of each T cell with endothelial cell-occupied space. T cells were tracked over time to measure velocity and total distance travelled. Measurements were calculated as an average of all tracked cells. Endothelial cells occupied 36.7 ± 0.8% of each image and there were 54.83 ± 1.9 lymphocytes within the field of vision at the start of each session. On average, lymphocyte tracking lasted 40.9 ± 0.63 minutes. The distance-based tracking algorithm was chosen based on object label continuity over training image sets. The average velocity and true displacement were used for each object.

### Statistical Analysis

Statistical analyses were performed using GraphPad Prism (GraphPad Software). The statistical significance of the data was determined using Wilcoxon tests, Paired t-tests or Mann-Whitney U tests as indicated (*p < 0.05, **p < 0.01, ***p < 0.001 and ****p < 0.0001).

## Results

### Inflammatory Conditions for HLA Class II Expression and Endothelial Activation

**Figure 1:**
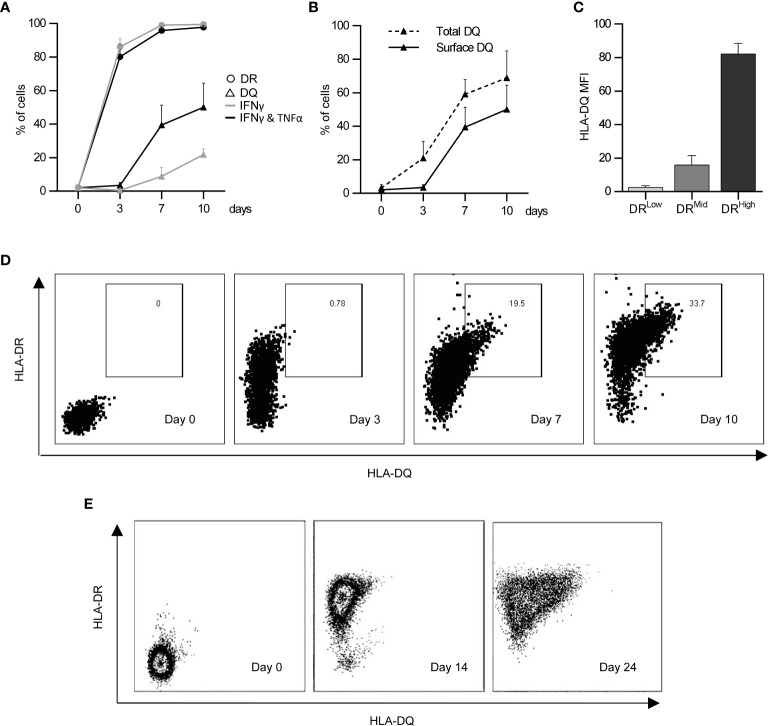
Induction of HLA-DQ protein expression by endothelial cells under inflammatory conditions. HLA-DR (circles) and HLA-DQ (triangles) expression was monitored over the course of 10 days in human microvascular endothelial cells (HMEC) stimulated either with IFNγ for 3 days (IFNγ; grey lines) or with IFNγ for 4 days followed by 3 days of IFNγ and TNFα (IFNγ & TNFα; black lines) (n = 3; mean +/- SEM) **(A)**. HLA-DQ detected at the cell surface (solid line) was compared to total HLA-DQ detected in permeabilised cells (dashed line) (n = 3; mean +/- SEM) **(B)**. The coexpression of HLA-DR and HLA-DQ was characterised in cells stimulated with IFNγ and TNFα as previously described at day 7 **(C)** (n = 3; mean +/- SEM). **(D)** shows a representative experiment examining co-expression of HLA-DR and HLA-DQ over a 10 day period in HMEC, whilst **(E)** shows a representative experiment examining HLA-DR and HLA-DQ expression by primary human renal glomerular endothelial cells after IFNγ and TNFα stimulation for 0, 14 and 24 days.

Endothelial cells do not express HLA class II molecules *in vitro* without continuous inflammatory stimulation (18). Previous studies (18, 23, 24) explored endothelial immunoregulation using activated dermal microvascular endothelial cells (aEC), which were exposed to IFNγ for 3 days to obtain significant cell surface HLA-DR expression (on 86.1 ± 3.5% of cells), yet these cells did not express HLA-DQ (**Figure 1A**). Longer exposure to IFNγ was required to induce HLA-DQ; 10 days of IFNγ stimulation induced HLA-DQ expression on only 21.8 ± 2.6% of cells. The combination of IFNγ and TNFα enhanced HLA-DQ expression earlier to produce highly activated endothelial cells (haEC), where 39.5 ± 8.3% of cells were HLA-DQ^+^ after 7 days (**Figure 1A**). HLA-DQ surface expression was delayed compared with intracellular expression (59.1 ± 6.4% HLA-DQ^+^ on day 7), potentially evoking inefficiency in HLA-DQ transport (**Figure 1B**) compared with HLA-DR (**Supplementary Figure 1**). It is noteworthy that HLA-DQ expression was limited to activate HLA-DR^high^ cells (**Figures 1C, D**).

The activated HLA-DR^+^ endothelial cells (aEC) and the highly activated HLA-DR^++^ HLA-DQ^+^ endothelial cells (haEC) were compared for their expression of molecules implicated in allorecognition and modulation of T lymphocyte responses (**Tables 1** and **2**). The haEC increased ICAM-1, required for adhesion and migration of lymphocytes (40) (haEC MFI 3111 ± 758.4 versus aEC MFI 646 ± 134.6; p<0.01) and decreased VE cadherin, which is important for endothelial cell junctions and permeability (40) (aEC MFI 741 ± 148 versus haEC MFI 570 ± 140.9; p<0.05). Highly activated endothelial cells increased their expression of the co-inhibitory molecule PD-L1 (aEC MFI 1768 ± 697 versus haEC MFI 2478 ± 896.6; p<0.05).

**Table 1:** Phenotype of endothelial cells activated under different conditions by pro-inflammatory cytokines.

		% cells (mean)			MFI (mean)		
*HMEC Phenotype*		aEC	haEC	*p-* value	aEC	haEC	*p-* value
*HLA antigens:*	HLA-DR	72	91	**	1870	5459	*
	HLA-DQ	2	29	****	98	559	*
	HLA-ABC	99	99	ns	4404	4118	ns
*Adhesion:*	ICAM-1	81	97	ns	646	3111	**
	VE Cadherin	89	86	ns	741	570	*
*Costimulation:*	PD-L1	77	82	*	1768	2478	*
*Complement regulation:*	CD59	99	99	ns	25159	12892	**
	CD55	99	98	ns	1175	1193	ns
	CD46	99	98	ns	5082	4808	ns

Microvascular endothelial cells were stimulated for 3 days with IFNγ (aEc, activated endothelial cells) or with 4 days of IFNγ followed by 3 days of IFNγ and TNFα (haEC, highly activated endothelial cells). Both the percentage of expressing cells and the mean fluorescence intensity of expression were determined (n ≥ 3; paired t test). ns, non-significant.

**Table 2:** Phenotype of primary renal glomerular endothelial cells activated under pro-inflammatory cytokines.

		MFI (mean)				
*Renal glomerular endothelial cells phenotype*		0	14	24 days	*0-14 days*	*14-24 days*
					*p-* value	*p-* value
*HLA antigens:*	HLA-DR	36.20	2783	4860	*	ns
	HLA-DQ	2.6	67	242	ns	**
	HLA-ABC	18802	28633	32584	ns	ns
*Adhesion:*	ICAM-1	455	15810	47449	**	***
*Costimulation:*	PD-L1	1923	6088	10777	**	**

Endothelial cells were continuously stimulated with with IFNγ and TNFα for 14 or 24 days. The mean fluorescence intensity of expression were determined (n ≥ 3; one-way ANOVA then Tukey’s post hoc comparisons).

Given the significance of complement activation in chronic rejection, the endothelial expression of complement regulatory molecules was analyzed following inflammatory stimulation. Expression of CD55 and CD46 were similar in aEC and haEC, whereas CD59 expression was reduced by almost half in haEC (**Supplementary Table 1**).

The lymphocyte-regulating phenotype induced by exposure to inflammatory conditions was also examined in primary renal glomerular endothelial cells. These cells were more resistant to the induction of HLA class II antigens and required longer periods of exposure to inflammatory cytokines. HLA-DR was detected after 7 days of continuous IFNγ stimulation, whilst HLA-DQ only became evident between 14 and 24 days of combined IFNγ and TNFα exposure (**Figure 1E**). Similarly, to HMEC cells, primary renal glomerular cells upregulated HLA-DR, HLA-DQ, ICAM-1 and PD-L1 with prolonged continuous inflammatory conditioning (**Supplementary Table 2**).

### Sustained Inflammation Alters the Endothelial Modulation of CD4^+^ T Cell Alloresponses

Allogenic human microvascular endothelial cells can selectively expand proinflammatory and anti-inflammatory T helper subsets: Th1, Th17 and memory Treg (18, 23, 24). To assess how the level of inflammation modulates endothelial cell alloregulation of CD4^+^ T immune responses, haEC and aEC were cultured with non-HLA-matched PBMC for 7 days before analysis of T cell proliferation and differentiation.

Memory T cell proliferation was modestly reduced (aEC 17.5% versus haEC 15.3%; p<0.05), despite higher expression of HLA class II on haEC compared with aEC (**Supplementary Figure 2A**). Expansion of pro-inflammatory Th1 and Th17 subsets was equivalent in cultures with aEC or haEC (**Supplementary Figures 2B, C**). The time course of detection of memory T cells, their proliferation and their apoptosis were examined and were comparable after co-culture with either aEC or haEC (**Supplementary Figures 2D, F**).

**Figure 2:**
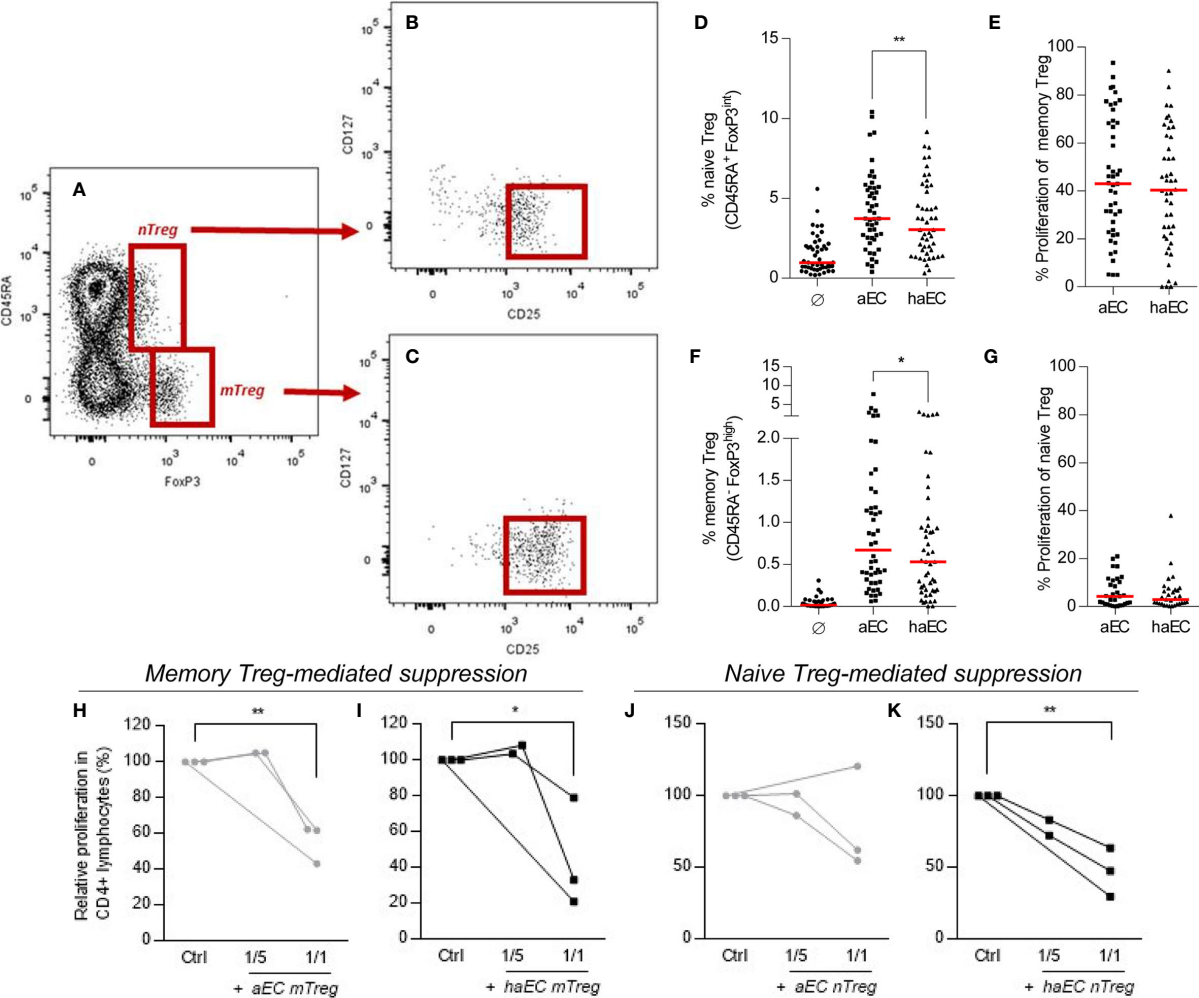
Human microvascular endothelial cells increase the fraction of naïve and memory regulatory subsets in CD4^+^ T lymphocytes. PBMC were cultured in the absence of endothelial cells (Ø), in the presence of activated endothelial cells (aEC) or highly activated endothelial cells (haEC). Naïve Treg were defined as CD4^+^ CD45RA^+^ FoxP3^+^ **(A)**; these cells were mostly CD127^-^ CD25^+^ **(B)**. Memory Treg were defined as CD4^+^ CD45RA^-^ FoxP3^++^ **(A)**; these cells were mostly CD127^-^ CD25^++^ **(C)**. The expansion of naïve and memory Treg subsets was assessed **(D, F)**, in addition to their respective proliferation during culture **(E, G)** (n = 48; red lines represent the median; Wilcoxon test). Memory and naïve Treg subsets were evaluated for their capacity to suppress the proliferation of responder CD4^+^ T lymphocytes. Memory and Naïve Treg (mTreg and nTreg) were isolated from 7-day cocultures with either activated or highly activated endothelial cells (aEC or haEC). Treg subsets were isolated by sorting from PBMC, then cultured with autologous responder CD4^+^ T lymphocytes and CD3/CD28 Dynabeads at a ratio of 1 or 5 responder CD4^+^ T cells per bead. The proportion of responders having undergone three divisions is represented as a percentage of the maximum proliferation in the absence of Treg cells. Memory Treg (mTreg) and naïve Treg (nTreg) from aEC cocultures are represented with grey lines **(H–K)**, whilst mTreg and nTreg from haEC cocultures are shown in black lines (**H–K**; n =3; paired t-test).

The proportion of memory Treg (CD4^+^ CD45RA^-^ FoxP3^++^) was increased after PBMC culture with aEC (**Figure 2F**), as previously described^18^ with an increase from a baseline level of 0.02 to 0.7% in aEC cultures (p<0.0001). Naïve Treg cells (CD4^+^ CD45RA^+^ FoxP3^+^) also increased from a baseline level of 1,0% to 3.7% (p<0.0001) in aEC cultures (**Figures 2A, B**). Both naïve and memory Treg were CD127^-^ CD25^+^ (**Figures 2A–C**). Yet, in contrast to pro-inflammatory Th17 and Th1, the Treg fraction was strongly reduced following co-culture with haEC. The proportion of naïve and memory Treg decreased by 19% (p<0.01) and by 21% respectively (p<0.05; **Figures 2D, F**).

The suppressive potential of memory Treg expanded by aEC has been demonstrated (18, 23). In order to compare Treg populations following culture with either aEC or haEC, their ability to suppress proliferation of autologous CD4^+^ T cells was determined. Treg expanded or induced in cultures with aEC or haEC had equivalent suppressive capacity (**Figures 2H–K**). On average, naïve or memory Treg reduced effector proliferation by 42% at a ratio of 1:1 (**Figures 2H–K**).

Naïve Treg did not proliferate (**Figure 2G**) and memory Treg proliferation was similar whether expanded by aEC or haEC (**Figure 2E**). The state of endothelial inflammation in cultures did not affect Annexin V detection or proliferation of mTreg over a time course of 0, 5 and 7 days (**Supplementary Figures 2D-F**), nTreg (data not shown) or the whole T memory cell population (**Supplementary Figure 2F**). Reduced survival was therefore not responsible for impaired expansion and/or induction of the proportion of Treg mediated by haEC and so we next explored other factors potentially implicated.

### Impaired Treg Expansion Is Not Associated With Known Soluble Factors

**Figure 3:**
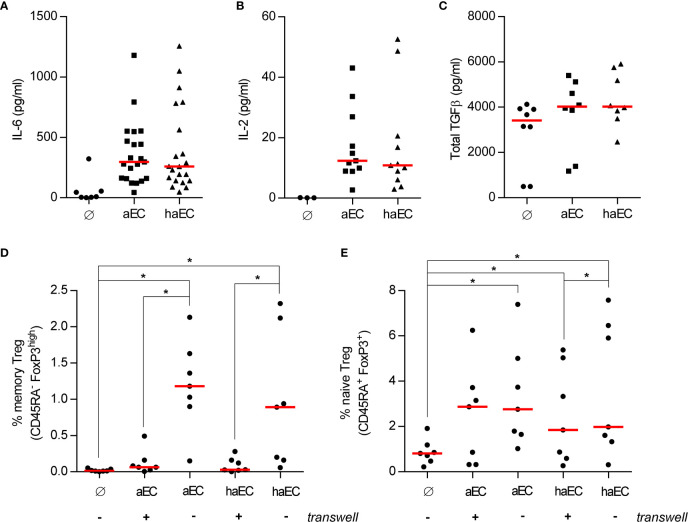
Soluble factors are important for the increased proportion of naïve Treg and not the cause of impaired endothelial-mediated Treg expansion. PBMC were cultured in the absence of endothelial cells (Ø), in the presence of activated endothelial cells (aEC) or highly activated endothelial cells (haEC). After 3 days, the culture supernatants were collected and analysed for the presence of the selected cytokines (medians; Wilcoxon test): IL-6 [**(A)**; n = 21], IL-2 [**(B)**; n = 11] and total TGFβ [**(C)**; n = 8]. The physical interaction between endothelial cells and PBMC was prevented by a porous cell culture insert (+ or -) and the expansion of memory [**(D)**; n = 7] or naïve Treg [**(E)**; n = 7] was assessed after 7 days (red lines represent the median; Wilcoxon test).

We have previously carried out multiplexed assays to identify which cytokines are produced in aEC-PBMC co-cultures and found that high amounts of IL-6 are secreted. IL-6 has been reported to antagonise peripheral Treg induction (17), whilst *in vitro* Treg induction is promoted by IL-2 and TGFβ (41). We examined IL-6, IL-2 and TGFβ levels in PBMC co-cultures and did not observe any difference between aEC or haEC cultures (**Figures 3A–C**). These data suggest equally permissive cytokine environments for Treg increases in response to aEC or haEC.

The requirement for direct contact in Treg increases was further examined by preventing the endothelial cell-PBMC interaction. Direct contact was indispensable to increase the memory Treg fraction (**Figure 3D**), whereas soluble factors alone were sufficient to increase naive Treg from 0.8% to 2.9% in aEC cultures (p<0.05; **Figure 3E**) or to 1.8% in haEC cultures. Soluble factors produced in aEC and haEC cultures were equally potent for naïve Treg induction (**Figure 3E**). These data highlight the importance of cell-cell interactions for optimal expansion of memory Treg.

### Endothelial PD-L1 Is Associated With Decreased Treg Expansion and Enhanced TCR Signaling

**Figure 4:**
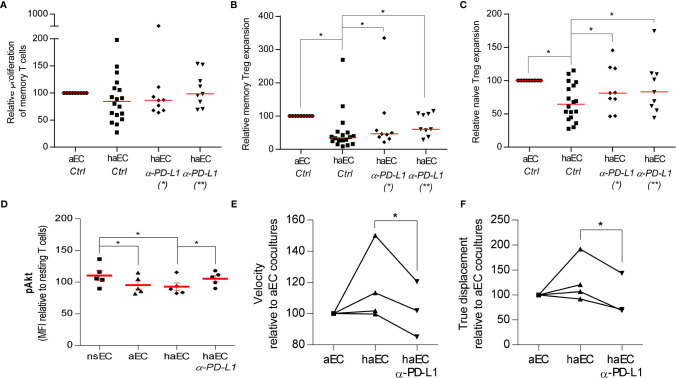
Increased Treg proportions by highly activated endothelial cells could be modulated by blocking PD-L1. PBMC were cultured with activated endothelial cells (aEC) or highly activated endothelial cells (haEC). haEC were pre-treated with 10µg/ml monoclonal blocking antibodies against PD-L1(*; n = 9) and washed or the blocking antibodies were added directly to the culture (*; n = 9). For comparison, aEC and haEC were untreated or treated with 10µg/ml non-specific IgG2b [Ctrl; n = 18. Untreated and IgG2b-treated endothelial cells did not differ in their capacity to induce alloproliferation or Treg increases. After 7 days of coculture, we determined the proliferation of memory T cells (CD4^+^ CD45RA^-^; **(A)**] and amplification of memory Treg [CD4^+^ CD45RA^+^ FoxP3^+^; **(B)**] and naïve Treg [CD4^+^ CD45RA^-^ FoxP3^++^; **(C)**] relative to the maximal expansion observed after culture with aEC (red lines represent the median, Wilcoxon test). Phosphorylation of Akt (pAkt) in CD4^+^ T cells was modified after exposure to activated endothelial cells. CD4^+^ T lymphocytes were cultured with non-stimulated endothelial cells (EC), activated endothelial cells (aEC), highly activated endothelial cells (haEC) or with haEC pre-treated with 10µg/ml monoclonal blocking antibodies against PD-L1 (haEC α-PD-L1) for 2 hours. Intracellular changes in pAkt at serine 473 [**(D)**; n = 5] were assessed. The mean fluorescence intensity (MFI) of each condition is calculated as a percentage of the MFI of resting CD4^+^ T cells in each experiment **(D)** (red lines represent the mean, paired t test). Lymphocyte motility was differentially modified by the state of activation in endothelial cells. CD4^+^ T lymphocytes were cultured with activated endothelial cells (aEC), highly activated endothelial cells (haEC) or with haEC pre-treated with 10µg/ml monoclonal blocking antibodies against PD-L1 (haEC α-PD-L1). The velocity of CD4^+^ T cells **(E)** and their displacement **(F)** were determined over 75 minutes of coculture. The mean measurements are relative to aEC values (n = 3; paired t test).

Changes in the direct interactions between haEC and PBMC may be associated with lessening Treg frequency. ICAM-1 and PD-L1 have been reported to play a role in T cell activation and Treg induction (18, 41). Both molecules are upregulated in haEC (**Table 1**), so blocking monoclonal antibodies (mAb) were used to elucidate their role. After exposure to a high level of inflammation, pre-incubation of haEC with 10µg/ml anti-ICAM-1 mAb did not alter the proportion of memory or naïve Treg (data not shown). Pre-incubation with 10µg/ml anti-PD-L1 mAb reduced the proportion of PD-L1^+^ cells detected by almost 50% (from 98% to 53%), producing a partial blockade of endothelial PD-L1 and resulted in almost doubling mTregs in haEC cultures (from 33% to 61% of the maximal aEC-mTreg expansion; p<0.05; **Figure 4B**). Naïve Treg induction was also increased (from 59% to 83% of the maximal aEC-nTreg expansion; p<0.05; **Figure 4C**). These increases in Treg were noted whether the anti-PD-L1 blocking antibody was only incubated with endothelial cells or was left throughout the co-culture. These data underline the differential effects of the PD-L1-PD-1 interaction according to the intensity of expression (42, 43).

PD-L1 engagement of PD-1 can regulate and antagonize TCR signaling (41, 44). Relative proliferation of memory T cells was equivalent in response to aEC or haEC and in the presence of the PD-L1 Ab (**Figure 4A**). To analyze the impact of inflammation and endothelial PD-L1 blockade on TCR signals, Akt phosphorylation at Ser473 (pAkt) was compared in primary CD4^+^- T cells from non-HLA matched donors in culture with EC. No differences were observed in pAkt after culture with aEC or haEC (**Figure 4B**). Despite high inter-individual variability in the phosphorylation response, PD-L1 blockade increased lymphocyte levels of pAkt (p<0.05; haEC 93 ± 6% versus PD-L1 blocked haEC 105 ± 4.9%), indicating enhanced TCR signaling (**Figure 4D**).

TCR signal transduction can induce migratory arrest, which may be observed using time-lapse microscopy (45–47). Having detected changes in T cell signal transduction, we determined whether T cell motility was altered. CD4^+^ T lymphocytes were tracked over a layer of endothelial cells. We examined T cell velocity (pixels per minute) as well as distance travelled from starting point or ‘true displacement’. These data measure lymphocyte migratory behaviour and indicate the quality of endothelial-lymphocyte interactions. No significant difference was observed in lymphocyte velocity or distance travelled in cultures with aEC or haEC (**Figures 4E, F**). However, PD-L1 blockade decreased both T lymphocyte velocity (p<0.05; 116 ± 11.67% versus 102 ± 10.24%) and displacement (p<0.05; 128 ± 22.13% versus 94 ± 24.6%; **Figures 4E, F**). Lymphocyte motility can therefore be modulated by endothelial PD-L1 expression. It is possible that limiting available PD-L1 may have reduced lymphocyte motility by enhancing the TCR-driven migratory arrest.

## Discussion

Microvascular endothelial cells have the capacity to amplify functional Treg subsets; this study and previous work indicates that the level of inflammation is a key determinant of whether the endothelium promotes or impairs anti-inflammatory responses (18).

The inflammatory conditions selected for endothelial activation replicated the upregulation of HLA-DR, HLA-DQ, ICAM-1 and PD-L1 observed during human allograft rejection (3–7, 48). Moreover, only a high level of inflammation requiring both IFNγ and TNFα replicated the *de novo* expression of HLA-DQ by microvascular endothelial cells post-transplantation. HLA-DR and HLA-DQ share the master class II transactivator (CIITA), yet their microvascular expression varies in quantity and context, *in vivo* and in this *in vitro* model (3–6). Intragraft inflammation may regulate allogenic HLA-DQ expression *in vivo* (49). Sustained inflammation also reduced expression of the CD59 complement regulatory protein, which inhibits the formation of membrane attack complexes (MAC) and prevents cell lysis. Downregulation of CD59 may be implicated in the sensitivity of the endothelium to complement-mediated cell activation.

Despite increased HLA-DR and -DQ expression, the highly activated endothelial cells did not increase the proportion of Treg compared with aEC but instead selectively decreased it. Because Treg resulting from culture with either aEC or haEC were functionally equivalent, the decreased proportion of Treg after endothelial exposure to a high level of inflammation therefore represents a real loss in immunosuppressive ability. There was also a disruption of the balance between Treg and pro-inflammatory CD4^+^-T because the proportion of Th17 or Th1 subsets was unchanged (**Supplementary Figure 2**). While memory Treg increases were associated with proliferation, this was not the case for naïve Treg therefore leading to the suggestion that they may be induced by soluble factors. Although we did not identify a direct role for IL-6, IL-2 or TGFβ, other soluble factors or indeed combinations of soluble factors may be implicated in Treg induction and/or maintenance. The suppressive activity of the Treg, both naïve and memory, was confirmed in order to exclude the possibility that FOXP3 expression was simply identifying transiently activated T cells.

In cocultures with aEC, endothelial ICAM-1 was required for the expansion and proliferation of memory Treg (18). ICAM-1 was also upregulated under conditions of high inflammation yet blocking ICAM-1 on the haEC did not alter memory Treg expansion. Highly activated EC may have a redundancy in ICAM-1 expression that does not negatively impact Treg expansion.

Highly activated EC simultaneously upregulated HLA class II antigens and the immunoinhibitory ligand, PD-L1. The PD-L1 molecule regulates T cell activation and is constitutively expressed by endothelial cells (44). PD-L1 mRNA is upregulated in renal allografts undergoing rejection (48) and endothelial PD-L1 protein expression is increased in murine models of cardiac allograft rejection (50, 51).

In our model, the blockade of endothelial PD-L1 coincided with increased expansion and/or induction of Treg. These results contrast with literature describing a role for PD-L1 in promoting Treg differentiation and function (41, 52). In mice, allogenic endothelial cells can expand Treg by a PD-L1-dependent mechanism (52). Under *in vitro* conditions for murine Treg induction from naïve CD4^+^ T cells, PD-L1 enhanced differentiation in a dose-sensitive manner. However at the highest concentrations, PD-L1 actually inhibited Treg induction and lymphocyte activation (41). This ambiguous role of PD-L1 is supported by *in vivo* models where strong PD-1/PDL-1 interactions impede the suppressive function of CD4^+^FoxP3^+^ Treg (42, 43). Select cancer patients who experience hyperprogressive disease after treatment with anti-PD-1 monoclonal antibodies were associated with intratumoral expansion of Treg; this human Treg proliferation could be replicated by PD-1 blockade *in vitro (* 53). The precedent for PD-L1 driven suppression of Tregs has been established in other models of chronic inflammation, such as human hepatitis virus C infection and murine graft versus host disease (54, 55). We do not exclude that a low level of PD-L1 may be beneficial to Treg expansion, yet PD-L1 expression by aEC was not implicated in *in vitro* human memory Treg expansion (18).

In this model of haEC, PD-L1 expressed by endothelial cells was active in regulating both the migratory speed and the distance travelled of allogeneic CD4^+^-T. These data may suggest that more lasting contacts with the EC are established when PD-L1 is not engaged. Treg development is sensitive to critical thresholds of TCR signals (15), therefore it is interesting that PD-L1 blockade coincided with increased differentiation of Treg. PD-L1 binding to the PD-1 receptor on lymphocytes is known to result in phosphatase recruitment and disruption of signaling by the TCR (44). PD-1 signaling reduces the phosphorylation of Akt (41, 44) and antagonizes T cell activation, proliferation and migratory arrest (13, 44, 45). After blocking endothelial PD-L1, we observed increased Akt phosphorylation and decreased lymphocyte motility despite high inter-individual variability in responses. Variability was expected given the use of primary non-HLA matched CD4^+^ T cells, which models the *in vivo* situation of alloreactivity. Given the changes in Akt phosphorylation following PD-L1 blockade, the impact of PD-L1 on Treg may be mediated by TCR signal regulation. PD-L1 may also have roles aside from TCR modulation since PD-L1 ligation has been recently implicated in endothelium activation and permeability (56).

Following organ transplantation the activation of the allograft endothelium is ultimately the result of numerous factors including: immunosuppressive therapies (24), feedback from naïve and effector immune cells (57, 58), complement activation (22), donor-specific antibody binding (23) and inflammation of the microvasculature is an independent determinant of renal allograft failure. This study revealed that the mechanisms of Treg amplification are more fragile under highly inflammatory conditions and that endothelial PD-L1 may have a nuanced role in Treg proliferation and/or induction during allotransplantation. Given the participation of inflammation in in other pathologies such as autoimmunity and cancer, the impaired generation of Treg by endothelial cells may have broad implications.

## Data Availability Statement

The original contributions presented in the study are included in the article/**Supplementary Material**. Further inquiries can be directed to the corresponding author.

## Ethics Statement

The studies involving human participants were reviewed and approved by Institutional Review Board of the Hôpital Saint Louis, Paris. Written informed consent for participation was not required for this study in accordance with the national legislation and the institutional requirements.

## Author Contributions

AC, JL, DG, and NM participated in designing the research. AC, JL, DG, and NM participated in analyzing the data and writing the paper. AC, JL, and KP participated in performing the research. All authors contributed to the article and approved the submitted version.

## Funding

AC was supported by the Société Francophone de Transplantation. INSERM and Vaincre le Mucoviscidose contributed to funding of this study.

## Conflict of Interest

The authors declare that the research was conducted in the absence of any commercial or financial relationships that could be construed as a potential conflict of interest.

## Abbreviations

EC, Endothelial cells; aEC, Activated endothelial cells; HLA, Human Leukocyte Antigen; HRGEC, Human Renal Glomerular endothelial cells; IFNγ, Interferon gamma; IL-6, Interleukin 6; PBMC, Peripheral Blood Mononuclear Cells; PD-L1, Programmed death-ligand 1; Akt, phosphoAkt; TGFβ, Transforming Growth Factor beta; TNFα, Tumor necrosis factor alpha; Treg, Regulatory T cell.
