# Supplementary figures and images for: Inflammation Determines the Capacity of Allogenic Endothelial Cells to Regulate Human Treg Expansion
Source: Front Immunol. 2021 Jul 9;12:666531. doi: 10.3389/fimmu.2021.666531 (PMC8299527; doi:10.3389/fimmu.2021.666531)

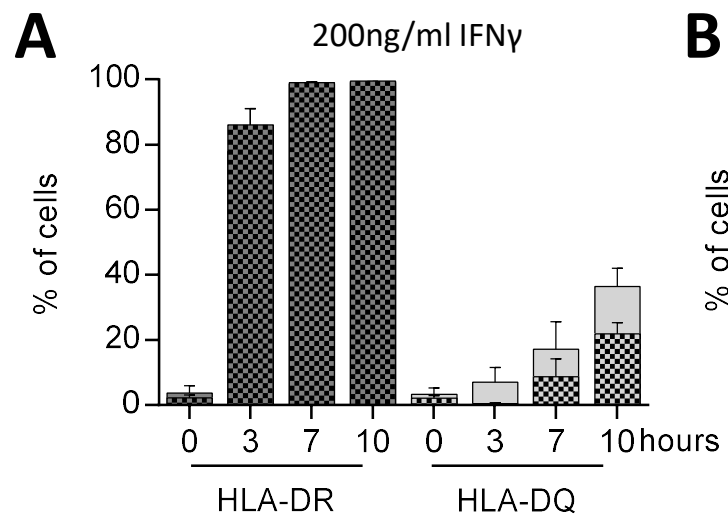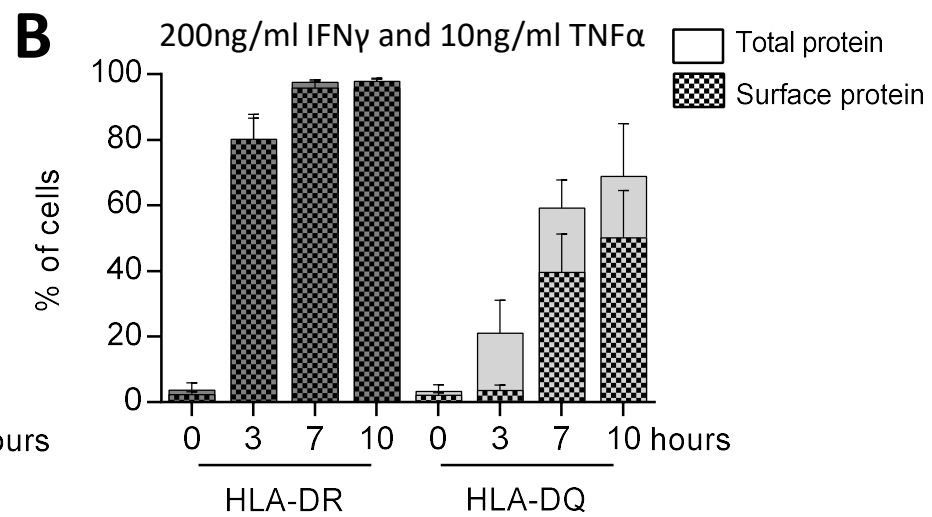

Supplement: Supplementary file 1 [file DataSheet_1.pdf]

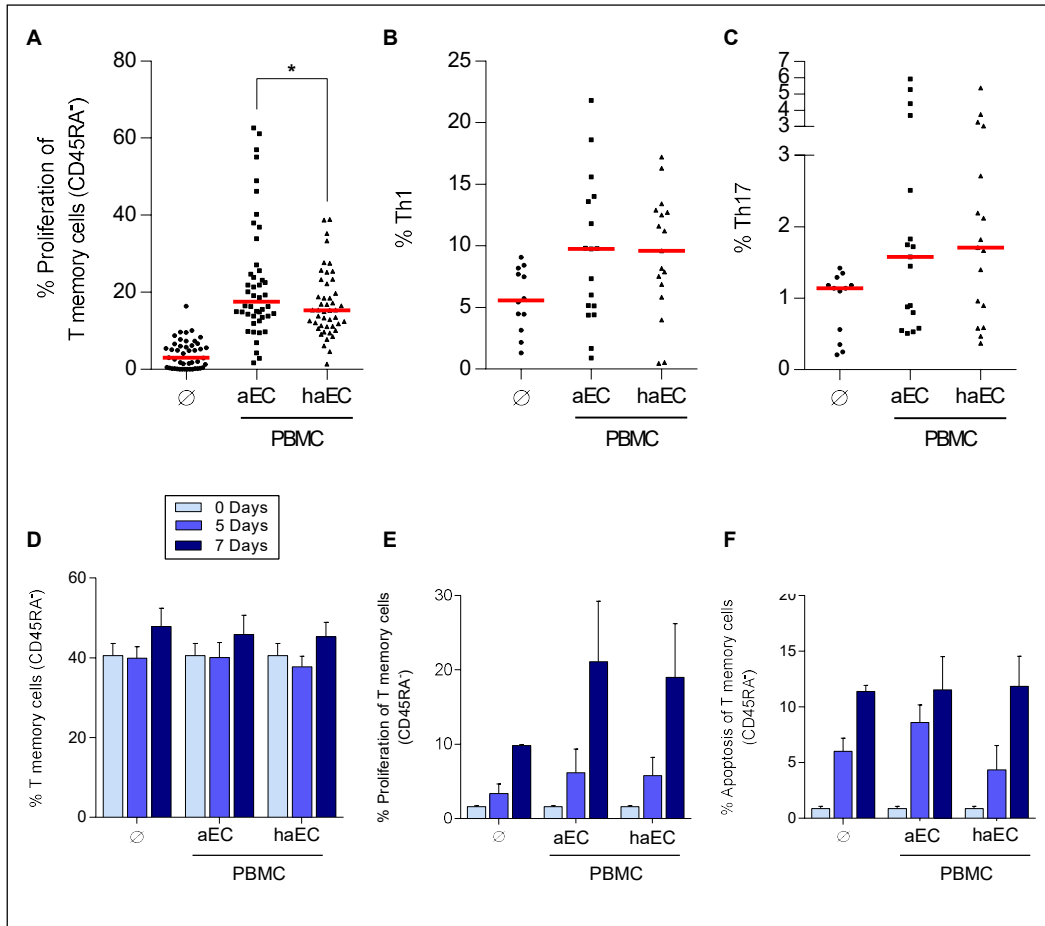

Supplement: Supplementary file 2 [file DataSheet_2.pdf]

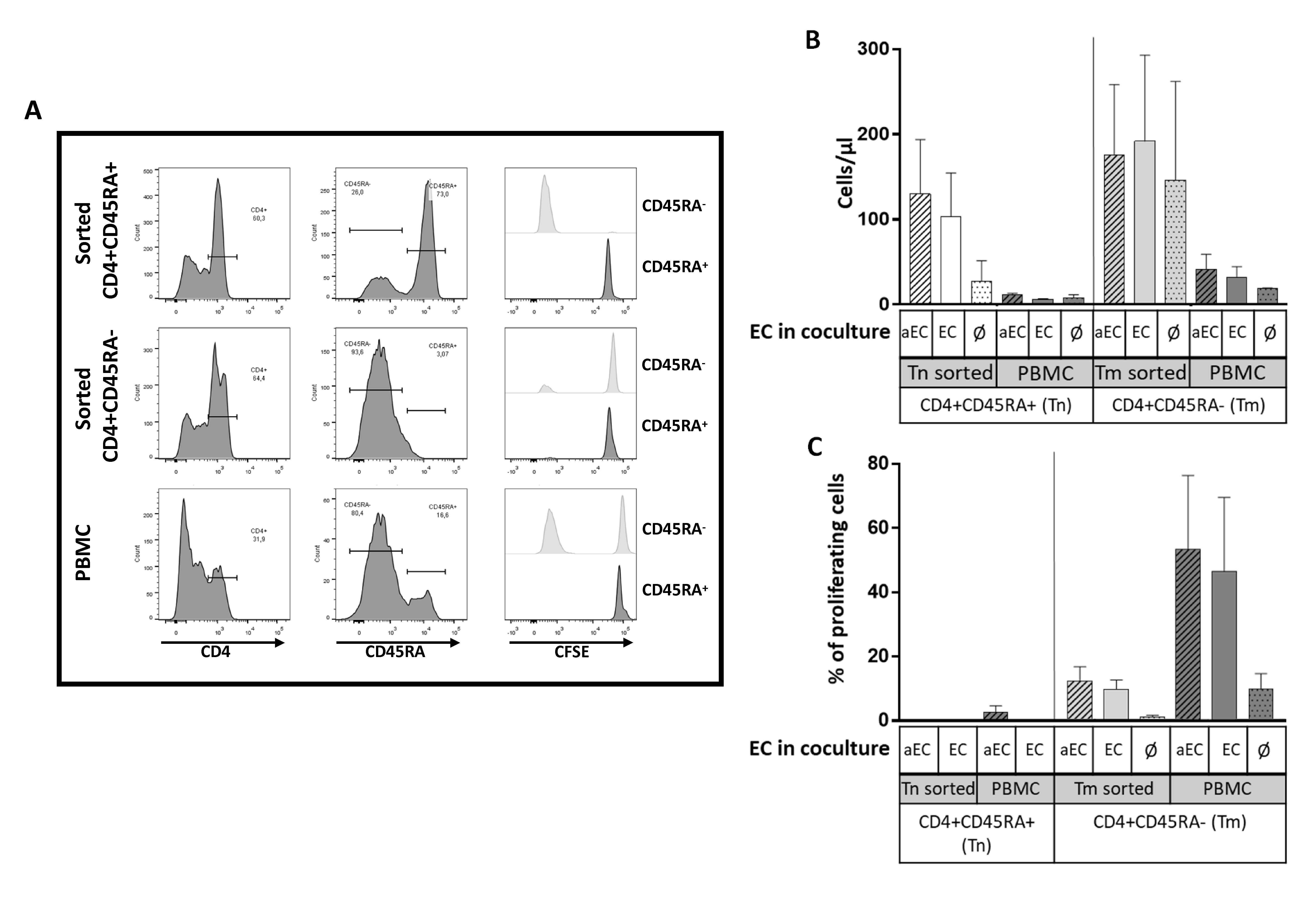

Supplement: Supplementary file 3 [file Image_1.tif]
